# ERRATUM: Hyperglycaemia-induced resistance to Docetaxel is negated by metformin: a role for IGFBP-2

**DOI:** 10.1530/ERC-16-0095e

**Published:** 2025-11-05

**Authors:** K M Biernacka, R A Persad, A Bahl, D Gillatt, J M P Holly, C M Perks

**Affiliations:** ^1^ IGFs & Metabolic Endocrinology Group, School of Clinical Sciences, Learning & Research Building, Southmead Hospital, Bristol, UK; ^2^ Department of Urology, Southmead Hospital, Bristol, UK; ^3^ Department of Clinical Oncology, Bristol Haematology and Oncology Centre, University Hospitals Bristol, Bristol, UK

The authors and journal apologise for an error in the above paper, which appeared in volume 24, part 1, 17–30 . The error relates to Fig. 5, given on page 24, in which the blots shown in panel F were not labelled correctly due to an inadvertent mix-up of blots. In panel F, the blot labelled ‘IGFBP-2’ should have been labelled as the ‘β-actin’ loading control, and the blot labelled ‘Tubulin’ should have been labelled as ‘IGFBP-2’. In addition, the last lane showing a positive β-actin control was missing from the ‘β-actin’ blot.

The blots in panel F relate to a cell line that was used as a negative control in which no changes were observed; therefore, this error has no impact on the results, interpretation, or conclusions of this paper.

For confirmation, the authors supplied uncropped full blots and the original laboratory record for review. The corrected Fig. 5 is given in full below:

**Figure 5:**
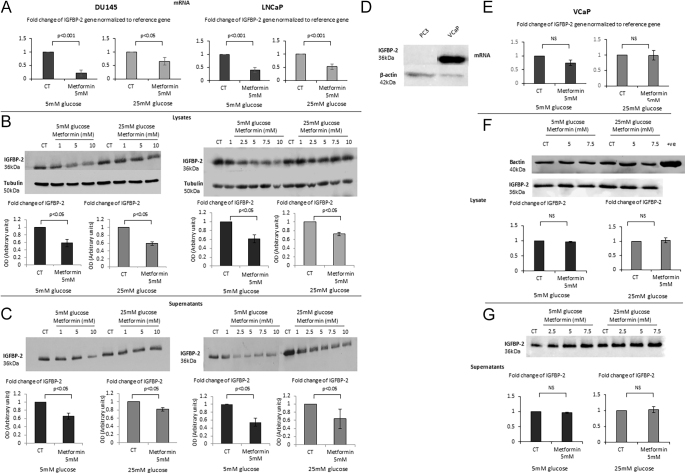
(A) Changes in mRNA levels of IGFB-2 in response to 5 mM metformin treatment in either 5 mM or 25 mM glucose conditions. DU145 and LNCaP cells were seeded at 0.8 × 10^6^ cells/T-25 flasks and cultured as described in Fig. 1A, mRNA was extracted 24 h after dosing with metformin, and Q-PCR was performed. Values of IGFBP-2 gene expression were normalised to the housekeeping gene (*18S*) (*n* = 3). (B) Western immunoblots show changes in the abundance of IGFBP-2 from DU145 or LNCaP cell lysates, respectively, exposed for 24 h to (1–10 mM) metformin in either 5 mM or 25 mM glucose. Cells were seeded at 0.5 × 10^6^ cells/T-25 flasks and cultured as described in Fig. 1A, and whole-cell lysates were collected and subjected to the Western blot technique. Each blot is representative of experiments repeated three times, and the densitometry shows the mean changes (*n* = 3). (C) Western immunoblot shows changes in the abundance of IGFBP-2 from DU145 cell supernatants exposed for 24 h to (1–10 mM) metformin in either 5 mM or 25 mM glucose. Cells were set up as described in Fig. 1A, and conditioned media were collected and subjected to the Western blot technique. Each blot is representative of experiments repeated three times, and densitometry shows the mean changes at a representative dose of metformin (*n* = 3). (D) Western immunoblot shows changes in the abundance of IGFBP-2 in PC3 and VCaP cells. (E) Changes in mRNA levels of IGFBP-2 in response to 5 mM metformin treatment in either 5 mM or 25 mM glucose conditions. VCaP cells were seeded at 0.8 × 10^6^ cells/T-25 flasks and cultured as described in Fig. 1A. mRNA was extracted 24 h after dosing with metformin, and Q-PCR was performed. Values of IGFBP-2 gene expression were normalised to the housekeeping gene (*18S*) (*n* = 3). (F) Western immunoblots show changes in the abundance of IGFBP-2 from VCaP cell lysates, respectively, exposed for 24 h to (5–7.5 mM) metformin in either 5 mM or 25 mM glucose. (G) Western immunoblot shows changes in the abundance of IGFBP-2 from VCaP cell supernatants exposed for 24 h to (2.5–7.5 mM) metformin in either 5 mM or 25 mM glucose.

